# Comparative Analysis of Readability Levels of Hand Surgery Patient Information on the ASSH Website and Artificial Intelligence

**DOI:** 10.1177/15589447261478323

**Published:** 2026-10-08

**Authors:** Neha R. Momin, Rosevine A. Azap, Zainab Yousaf, Addi Moya, Daniel P. Donato

**Affiliations:** 1 The University of Texas Medical Branch, Galveston, USA

**Keywords:** artificial intelligence, hand surgery, health education, patient education, readability

## Abstract

**Background:**

The American Medical Association (AMA) recommends patient education materials (PEMs) be written at a sixth-grade level to ensure accessibility. This study aims to compare hand surgery topics from the American Society for Surgery of the Hand (ASSH) website and artificial intelligence (AI)-generated PEMs to evaluate their alignment with the AMA’s recommendations.

**Methods:**

Ninety-eight hand surgery topics from the ASSH Hand Care “The Upper Extremity Expert” website were analyzed. Artificial intelligence (AI) platforms (Google Gemini 2.0 Flash and ChatGPT-4) were asked to: (1) create original PEMs based on ASSH website topics; (2) rewrite the ASSH topics at a sixth-grade reading level; and (3) create original PEMs over the ASSH topics at a sixth-grade reading level. Readability was assessed using the Flesch Reading Ease (FRE) and Flesch-Kincaid Grade Level (FKGL) scores.

**Results:**

The ASSH website and AI-generated PEMs initially exceeded the sixth-grade readability level. Original ASSH PEMs ranged from a readability of 10th to 12th-grade (FRE 57.5 [6.6]; FKGL 9.4 [1.2]). Google Gemini and ChatGPT’s initial PEMs were at the college level (Google Gemini: FRE 49.7 [7.5]; FKGL 9.6 [1.1] vs ChatGPT: FRE 47.4 [9.1]; FKGL 10.4 [1.4]). Artificial intelligence–rewritten ASSH content had improved readability, and AI-generated PEMs at a sixth-grade readability had lower word counts, higher FRE, and lower FKGL than original ASSH PEMs (all *P* < .001).

**Conclusions:**

Artificial intelligence platforms can be used as a tool to create hand surgery PEMs at the recommended sixth-grade reading level. Future efforts are needed to ensure the accuracy of AI-generated PEMs and accessibility to patients of varying health literacy for informed decision-making.

## Introduction

Despite extraordinary advances in our health care system and technologies around the world, the health literacy of many communities has continued to remain an area of global concern. It is estimated that approximately 80 million people in the United States have poor health literacy.^1^ This is alarming as low health literacy has been associated with poor patient outcomes, increased mortality, and increased health care costs.^2-5^ A patient’s health literacy should also be taken into account when providing patient education materials (PEMs), particularly in specialized fields such as hand surgery. The average American adult is estimated to read at an eighth-grade reading level.^6,7^ Given this limitation, the American Medical Association (AMA) recommends that PEMs be written at a sixth-grade reading level or below to improve patient comprehension.^8-11^ However, numerous studies have indicated that most PEMs, including in plastic surgery and hand surgery, exceed this recommended readability limits and are well above the average reading level of most American adults.^12-17^ This misalignment raises concerns that more accessible health communication strategies and PEMs are needed to ensure that patients are well informed about their health and health care.

Artificial intelligence (AI) has become routinely used in the common household since the launch of ChatGPT in 2022. Since then, several AI platforms have been released; however, Google Gemini has been particularly notable as it has been incorporated in Google’s search engine. Over the last several years, AI has been emerging in the health care field as a new tool that can be used to improve the care of patients in a myriad of ways. Given the popularity of these AI platforms, they can be leveraged as alternative tools for patients to explore health concerns and receive additional information. Very few studies have evaluated the utilization of AI and the generation of PEMs in a variety of specialties.^18-21^ These studies note the large potential for AI to enhance patient education; however, there is a paucity of data regarding the utilization of AI to regenerate hand surgery PEMs.

As patients are using online resources more to seek education about their health and health care, online PEMs are becoming increasingly important for patients to make informed and educated decisions regarding their health care. To this end, the purpose of our study was to compare hand surgery prompts on the American Society for Surgery of the Hand (ASSH) website and AI platforms (Google Gemini and ChatGPT) to evaluate their alignment with the AMA’s recommendations for readability of PEMs and investigate the utility of AI platforms for creation of hand surgery PEMs.

## Methods

### AI and ASSH Hand Care “The Upper Extremity Expert” Website PEMs

Information on 98 hand surgery topics was obtained from the ASSH Hand Care “The Upper Extremity Expert” website.^22^ Resources were evaluated in the English language only. Artificial intelligence platforms, ChatGPT-4, and Google Gemini 2.0 Flash were asked to: (1) rewrite the PEMs on the ASSH website at a sixth-grade reading level; (2) create original PEMs on the same topics from the ASSH website; and (3) generate original PEMs at a sixth-grade reading level. The prompts used were: (1) “Write patient education material over ‘(ASSH article title);’” (2) “Rewrite this at a 6th-grade or lower reading level: (paste ASSH article);” and (3) “Write patient education material over ‘(ASSH article title)’ at a 6th-grade or lower reading level.” These prompts were inputted using a new Google account and incognito browser. Subsequently, the previous chat was deleted, and a new chat was opened for each prompt for all of the 98 hand surgery topics.

### Scoring Modalities

The Flesch Reading Ease (FRE) and Flesch-Kincaid Grade Level (FKGL) scales are both validated readability formulas for the English language that were used to evaluate the reading grade level of the information that each PEM source provided.^23,24^ Both scales use sentence structure and textual complexity in their respective formulas to compute a readability score that correlates to varying levels of reading comprehension (Figure 1). The FRE scoring system ranges from 0-10 (Professional) to 100 (Fifth Grade). The FKGL scoring paradigm ranges from 0-3 (Kindergarten) to 15-18 (Postgraduation). For example, pertaining to the FKGL scoring system, an FKGL of 8 means that the reader would need an eighth-grade reading level or higher to comprehend the material. Per the AMA, PEMs are recommended to be at, or below a sixth-grade reading levels, which corresponds to an FRE score between 81 and 100 (fifth-grade and sixth-grade reading level) and an FKGL between 0 and 6 (sixth-grade reading level and below).^8-11^ The word count and readability scores of the ASSH website prompts and each newly generated PEMs were generated.

**Figure 1.**
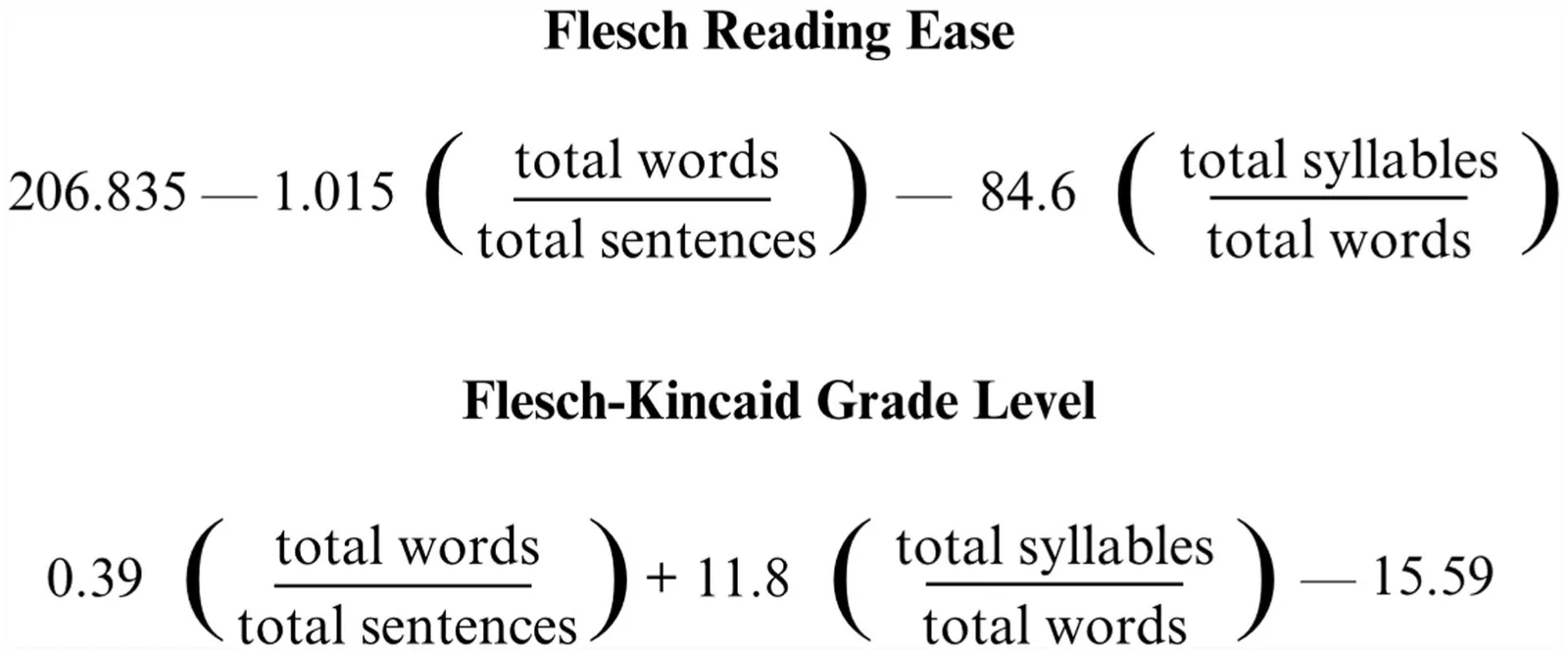
Flesch Reading Ease and Flesch-Kincaid Grade Level formulas.

### Statistical Analysis

Descriptive statistics were calculated for word count, FRE, and FKGL scores and are reported as mean with standard deviation. Linear mixed-effects models were used to compare scores across source conditions while accounting for repeated measurements of the same topics. Bonferroni-adjusted post hoc pairwise comparisons were performed when overall differences were significant. Residual Q-Q plots and histograms demonstrated approximate normality without severe departures; therefore, linear mixed-effects models were retained as the primary analytic approach. Statistical significance was set at *P* < .05. Analyses were performed using IBM SPSS Statistics.

## Results

All PEMs taken directly from the ASSH website were written above the recommended sixth-grade reading level, with a mean FRE score of 57.5 (SD = 6.6) and FKGL score of 9.4 (SD = 1.2) (Table 1). Original PEMs generated by Google Gemini had significantly shorter materials than both ASSH and ChatGPT, with a mean word count of 642.6 (SD = 75.7) (*P* < .001). Flesch Reading Ease scores differed significantly across all 3 groups, with ChatGPT producing the lowest FRE score at 47.4 (SD = 9.1), followed by Google Gemini at 49.7 (SD = 7.5), and ASSH at 57.5 (SD = 6.6) (*P* < .001). There was no significant difference between the FKGL score of ASSH and Google Gemini; however, ChatGPT produced material with a significantly higher FKGL score than both ASSH and Google Gemini (mean = 10.4; SD = 1.4) (*P* < .001) (Table 1).

**Table 1.** Readability Scores and Word Counts for Original ASSH and AI-Written PEMs.

Measure	ASSH Mean (SD)	Google Gemini Mean (SD)	ChatGPT Mean (SD)	*P* value
Word count	816.9 (400.5)^a^	642.6 (75.7)^b^	742.1 (86.0)^a^	<.001
FRE score	57.5 (6.6)^a^	49.7 (7.5)^b^	47.4 (9.1)^c^	<.001
	10th-12th grade	College	College	
FKGL score	9.4 (1.2)^a^	9.6 (1.1)^a^	10.4 (1.4)^b^	<.001
	High school	High school	High school	

Values are mean (standard deviation). Superscript letters denote Bonferroni-adjusted pairwise comparisons; values sharing a letter within a row are not significantly different. Overall *P* values are from linear mixed-effects models.

Abbreviations: AI, artificial intelligence; ASSH, American Society for Surgery of the Hand; FKGL, Flesch-Kincaid Grade Level; FRE, Flesch Reading Ease; PEM, patient education material; SD, standard deviation.

When Google Gemini and ChatGPT were prompted to rewrite the ASSH articles at, or lower than a sixth-grade reading level, both AI platforms were able to rewrite the ASSH articles at a lower reading level (FRE: 8th and 9th grade; FKGL: middle school) as compared to the original reading level of the ASSH articles (FRE: 10th-12th grade; FKGL: high school) (*P* < .001) (Table 2). The mean FRE scores did not differ significantly between Google Gemini and ChatGPT rewrites; however, Google Gemini produced a significantly lower mean FKGL score than ChatGPT (*P* < .001). Furthermore, the word count for Google Gemini rewrites (mean = 1,792.4; SD = 646.0) was significantly higher as compared to ASSH (mean = 816.9; SD = 400.5) and ChatGPT rewrites (mean = 895.0; SD = 594.1) (*P* < .001) (Table 2).

**Table 2.** Readability Scores and Word Counts for Original ASSH PEMs and Those Rewritten by AI Platforms.

Measure	ASSH Mean (SD)	Google Gemini Mean (SD)	ChatGPT Mean (SD)	*P* value
Word count	816.9 (400.5)^a^	1792.4 (646)^b^	895 (594.1)^a^	<.001
FRE score	57.5 (6.6)^a^	68.8 (6.3)^b^	68.0 (7.9)^b^	<.001
	10th-12th grade	8th and 9th grade	8th and 9th grade	
FKGL score	9.4 (1.2)^a^	7.2 (1.0)^b^	7.6 (1.3)^c^	<.001
	High school	Middle school	Middle school	

Values are mean (standard deviation). Superscript letters denote Bonferroni-adjusted pairwise comparisons; values sharing a letter within a row are not significantly different. Overall *P* values are from linear mixed-effects models.

Abbreviations: AI, artificial intelligence; ASSH, American Society for Surgery of the Hand; FKGL, Flesch-Kincaid Grade Level; FRE, Flesch Reading Ease; PEM, patient education material; SD, standard deviation.

When the AI platforms were prompted to generate their own original PEMs at a sixth-grade or lower reading level, Google Gemini (mean = 459.4; SD = 54.1) and ChatGPT (mean = 463.5; SD = 285.4) had statistically significant lower word counts as compared to the original ASSH prompts (mean = 816.9; SD = 400.5) (*P* < .001) (Table 3). On average, Google Gemini produced the highest FRE score with a mean of 81.9 (SD = 6.8), followed by ChatGPT with a mean FRE score of 78.8 (SD = 6.8), both of which were significantly higher than ASSH (*P* < .001). Both Google Gemini (FKGL mean = 4.4; SD = 1.0) and ChatGPT (FKGL mean = 5.4; SD = 1.0) produced PEMs that were at an elementary school level of comprehension, whereas the original ASSH material was at a high school readability level (*P* < .001).

**Table 3.** Readability Scores and Word Counts for Original ASSH PEMs and AI-Written PEMs at a Sixth-Grade or Lower Reading Level.

Measure	ASSH Mean (SD)	Google Gemini Mean (SD)	ChatGPT Mean (SD)	*P* value
Word count	816.9 (400.5)^a^	459.4 (54.1)^b^	463.5 (285.4)^b^	<.001
FRE score	57.5 (6.6)^a^	81.9 (6.8)^b^	78.8 (6.8)^c^	<.001
	10th-12th grade	6th grade	7th grade	
FKGL score	9.4 (1.2)^a^	4.4 (1.0)^b^	5.4 (1.0)^c^	<.001
	High school	Elementary	Elementary	

Values are mean (standard deviation). Superscript letters denote Bonferroni-adjusted pairwise comparisons; values sharing a letter within a row are not significantly different. Overall *P* values are from linear mixed-effects models.

Abbreviations: AI, artificial intelligence; ASSH, American Society for Surgery of the Hand; FKGL, Flesch-Kincaid Grade Level; FRE, Flesch Reading Ease; PEM, patient education material; SD, standard deviation.

The mean FKGL scores of both the ASSH website and AI-generated PEMs are shown in Figure 2. The original ASSH and AI-generated PEMs were above the AMA-recommended sixth-grade reading level and above the eighth-grade average American adult reading level. The average hand surgery PEMs were found to be below the eighth-grade average American adult reading level when AI was prompted to rewrite the ASSH prompts at a sixth-grade or lower reading level; however, the results were still above the standard sixth-grade level. Hand surgery PEMs were only found to be below the recommended sixth-grade reading level when AI platforms were prompted to write original PEMs at or below a sixth-grade level.

**Figure 2.**
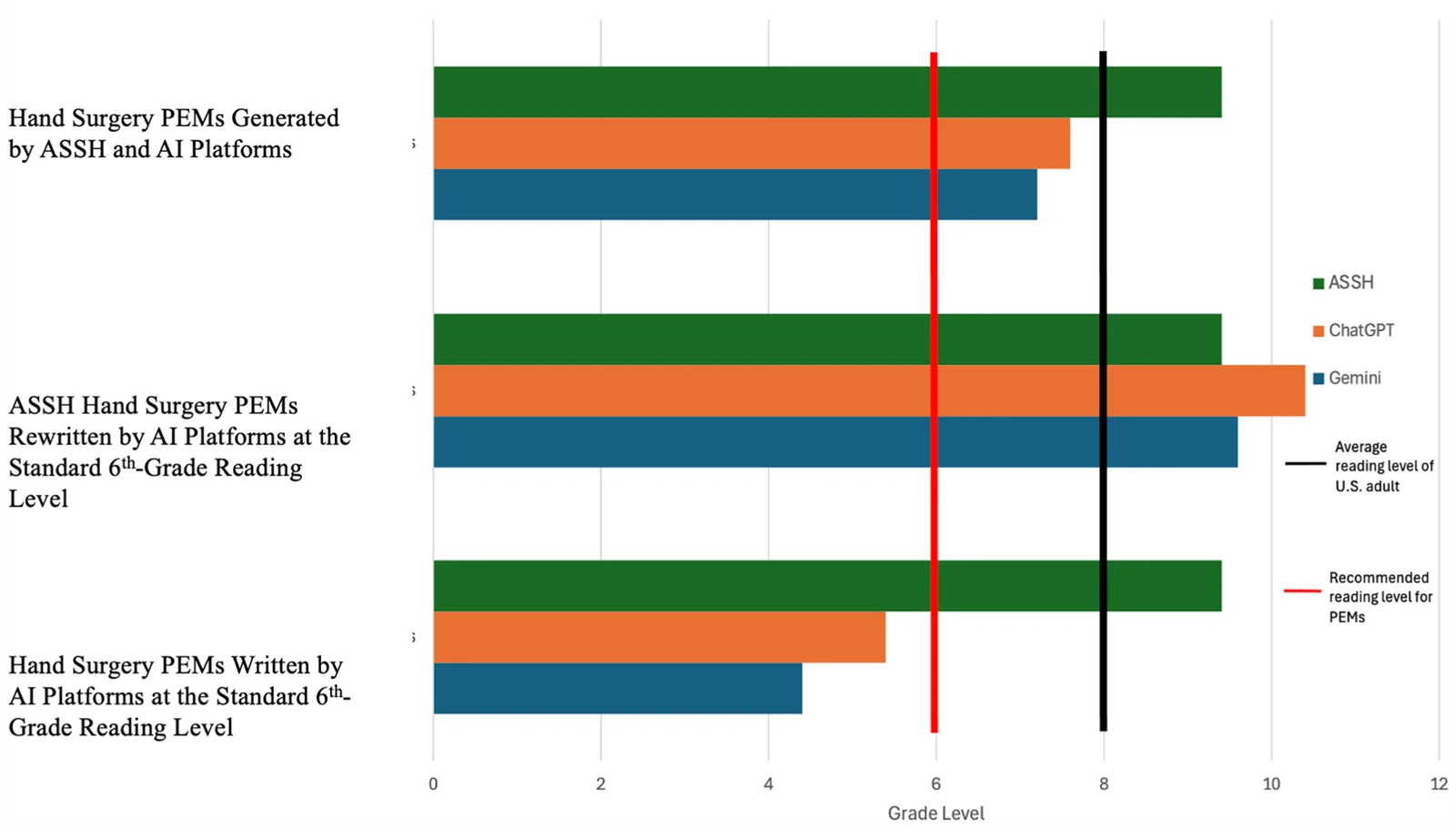
Mean grade level of online PEMs found on each platform using Flesch-Kincaid Grade Level (FKGL) readability scale. AI indicates artificial intelligence; ASSH, American Society for Surgery of the Hand; PEMs, patient education materials.

## Discussion

Over the years, our health care system has recognized the vital importance of social determinants of health and the implications they have on one’s health. One of these factors is health literacy, which, if poor, has been shown to be negatively correlated to poor health outcomes and rising health care costs.^2,8,10,25,26^ Thus, it is of upmost importance to health care providers to ensure that PEMs are at a readability that ensures patient comprehension so patients may make informed decisions regarding their health and health care. The average American adult reads at approximately an eighth-grade reading level.^6,7^ In addition, the AMA recommends for PEMs to be written at a sixth-grade reading level.^8-11^ Hand surgery, in particular, is a very specialized field that patients may not have a wide breadth of prior exposure to. For this reason, patients may elect to use various online resources to educate themselves on various hand conditions. In fact, studies have shown that patients will seek health information from the internet before and after their consultation with a physician.^27,28^

Our study demonstrated that the current PEMs on the ASSH website and the PEMs originally generated by the AI platforms are above the AMA-recommended sixth-grade reading level. This is important because patients use these sites to better understand their health conditions and subsequently make health care decisions. The extant literature supports our findings, particularly with ophthalmology and trauma surgery, noting the prevalence of suboptimal readability of PEMs.^12,29-31^ Our findings also align with previous studies conducted on hand and upper extremity PEMs. Wang et al^32^ evaluated the readability of articles on both the ASSH and American Academy of Orthopaedic Surgeons (AAOS) websites and found an average FKGL of 10.4 and 8.5, respectively. Furthermore, Eberlin et al^33^ evaluated the readability of carpal tunnel syndrome on 13 of the most popular website articles. The authors found an average FKGL of 12.6, which is well above a sixth-grade readability. Notably, Shin et al^34^ demonstrated similar findings as they found that the online PEMs in English and Spanish for polydactyly and syndactyly were above the recommended reading standards. These findings suggest that PEMs, even on professional society websites, are often written above the AMA-recommended sixth-grade readability. This is of importance as health care providers are likely to refer patients to these society webpages as the providers may feel that they will provide the most accurate information.

In contemporary society, there has been an increasing rise in the use of AI platforms in various facets. Notably, AI has become increasingly accessible, and patients have the ability to ask health and health care–related questions to these new AI platforms. Thus, it is important to assess whether the information generated by these AI platforms can be easily read and understood by the patients. Our study demonstrated that the initial PEMs generated by AI platforms were well above the recommended sixth-grade reading level; however, when specifically prompted to generate PEMs at a sixth-grade level, there were improvements. We found that Google Gemini, on average, was able to produce a readability of sixth-grade, and ChatGPT was able to produce a readability of approximately seventh grade. This demonstrates that AI platforms may have a pivotal role in the generation of PEMs if used correctly. Previous work by Moons and Bulck^35^ sought to use ChatGPT and Google Bard to improve the readability of 3 PEMs in scientific journals. The authors found that ChatGPT was able to improve the readability of the PEMs and decrease the word count; however, it was not able to achieve the sixth-grade reading standard. This was in contrast to Google Bard which was able to achieve the sixth-grade reading standard, however omitted critical information. Likewise, Kirchner et al^36^ found that ChatGPT could be successfully used to reduce complex PEMs to a sixth-grade reading level and below. Interestingly, this study also found that the PEMs generated by AI were both consistent and accurate. These studies highlight what may be a key aspect of AI’s emerging role in the health care system.

Several limitations should be considered when interpreting the results of our study. A key limitation is that the accuracy of AI-generated PEMs were not assessed. Readability alone does not guarantee the reliability or relevance of the medical information, and future studies should address this limitation by evaluating the accuracy of the AI-generated PEMs. Specifically, the factual accuracy, completeness, and potential for misinformation in AI-generated PEMs should be analyzed. This includes assessing whether educational materials generated by AI aligns with current evidence-based guidelines, omit clinically important information, or present inaccurate, misleading, or oversimplified recommendations that could adversely influence patient understanding and decision-making. Currently, there exist very few studies that have evaluated this. In addition, consistency of the PEMs generated by AI was not assessed. This study was also only conducted on PEMs in the English language, which limits the generalizability of our study. Furthermore, while FRE and FKGL scores are widely used to assess readability, they may overestimate difficulty scores by penalizing longer, but easily understandable words, potentially skewing the results. Similarly, FRE and FKGL scores do not consider document factors, such as photos and charts, and person factors, such as health literacy and education level, which influence one’s comprehension of a given text. Last, the use of free AI platforms limits the generative capability of these tools.

## Conclusions

In conclusion, AI platforms can be used as a tool to create PEMs at the recommended sixth-grade reading level, as compared to other widely available online PEMs. The available online PEMs on hand surgery topics currently are far too advanced for the comprehension of the average American patient. More work is needed to ensure that PEMs generated by AI are accurate and accessible to patients of a variety of health literacy proficiency levels so that patients can make informed decisions regarding their health and health care.

## Supplemental Material

sj-docx-1-han-10.1177_15589447261478323 – Supplemental material for Comparative Analysis of Readability Levels of Hand Surgery Patient Information on the ASSH Website and Artificial Intelligence Supplemental material, sj-docx-1-han-10.1177_15589447261478323 for Comparative Analysis of Readability Levels of Hand Surgery Patient Information on the ASSH Website and Artificial Intelligence by Neha R. Momin, Rosevine A. Azap, Zainab Yousaf, Addi Moya and Daniel P. Donato in HAND
