# Supplementary material for: Comparative Analysis of Readability Levels of Hand Surgery Patient Information on the ASSH Website and Artificial Intelligence
Source: Hand (N Y). 2026 Oct 8:15589447261478323. Online ahead of print. doi: 10.1177/15589447261478323 (PMC13650052; doi:10.1177/15589447261478323)
Supplement: Supplementary file 1 [file sj-docx-1-han-10.1177_15589447261478323.docx]

**Supplemental Table S1.** FRE and FKGL scales and the respective reading levels

| Flesch Reading Ease Score (FRE) | 100-90 | 5th grade |
| --- | --- | --- |
|  | 90-80 | 6th grade |
|  | 80-70 | 7th grade |
|  | 70-60 | 8th and 9th grade |
|  | 60-50 | 10th to 12th grade |
|  | 50-30 | College |
|  | 30-0 | College Graduate |
| Flesch-Kincaid Grade Level (FKGL) | 0-3 | Kindergarten/Elementary |
|  | 3-6 | Elementary |
|  | 6-9 | Middle School |
|  | 9-12 | High School |
|  | 12-15 | College |
|  | 15-18 | Post-graduate |

**Supplemental Table S2.** Word count, FRE, and FKGL scores of the original ASSH website articles and those edited by ChatGPT and Google Gemini to be written at a 6^th^-grade or lower reading level.

|  |  | **ASSH: Original Article from Website** | | | **Gemini: "Rewrite this at a 6th grade or lower reading level: (paste ASSH original)** | | | **ChatGPT: "Rewrite this at a 6th grade or lower reading level: (paste ASSH original)** | | |
| --- | --- | --- | --- | --- | --- | --- | --- | --- | --- | --- |
|  | **Article Title** | **Word Count** | **FRE** | **FKGL** | **Word Count** | **FRE** | **FKGL** | **Word Count** | **FRE** | **FKGL** |
| 1 | Amputation: Prosthetic Hand and Fingers | 1716 | 49.1 | 11 | 3093 | 61.8 | 8.5 | 636 | 74.9 | 6.7 |
| 2 | Animal Bite | 625 | 55.6 | 10.2 | 1694 | 68.8 | 7.3 | 534 | 74.4 | 6.7 |
| 3 | Antibiotics in Hand Surgery | 707 | 44.4 | 11.2 | 1740 | 53.5 | 9.2 | 540 | 53.4 | 9.2 |
| 4 | Arm Cast and Splint Care | 741 | 69.8 | 7.2 | 1843 | 81.4 | 5 | 552 | 84.2 | 4.6 |
| 5 | Arm, Hand, and Finger Replantation | 1134 | 57.4 | 9 | 2522 | 67.4 | 7.1 | 782 | 69.1 | 7.2 |
| 6 | Boutonniere Deformity | 348 | 65.3 | 7.7 | 921 | 73.8 | 6.1 | 613 | 70.2 | 6.8 |
| 7 | Boxer’s Fracture | 296 | 63.5 | 8.3 | 769 | 75.3 | 6.1 | 529 | 70.6 | 7.3 |
| 8 | Brachial Plexus Injury | 535 | 51.7 | 11.4 | 1497 | 68.2 | 8.1 | 1035 | 60.5 | 10.1 |
| 9 | Broken Arm | 493 | 61.3 | 9.9 | 1297 | 74.8 | 6.6 | 877 | 68.1 | 8.1 |
| 10 | Broken Finger Tip | 368 | 71.3 | 7.1 | 1051 | 80.2 | 5.4 | 697 | 75.1 | 6.6 |
| 11 | Broken Hand | 453 | 65.5 | 8.6 | 1142 | 79.9 | 5.1 | 829 | 73 | 7 |
| 12 | Carpal Boss | 376 | 63.4 | 8.8 | 993 | 76.2 | 6.3 | 684 | 70.3 | 7.7 |
| 13 | Carpal Tunnel Syndrome | 1973 | 52.3 | 10.6 | 3270 | 61.7 | 8.5 | 2510 | 55.4 | 9.9 |
| 14 | Cigarettes and Hand Conditions | 794 | 53.3 | 10.5 | 1724 | 66 | 7.9 | 1368 | 60.9 | 9.2 |
| 15 | Cold Hands | 541 | 61.5 | 9.8 | 1426 | 77.5 | 5.6 | 970 | 69.2 | 7.5 |
| 16 | Complex Regional Pain Syndrome - CRPS | 876 | 45.5 | 11.1 | 1961 | 58.7 | 8.7 | 1427 | 51.8 | 10.1 |
| 17 | Congenital Hand Differences | 1185 | 47.7 | 12.3 | 1945 | 53.7 | 10.7 | 1715 | 51.6 | 11.2 |
| 18 | Congenital Trigger Thumb | 499 | 63.3 | 9.4 | 1247 | 76.4 | 6 | 953 | 71.5 | 7.4 |
| 19 | Cortisone Shot | 294 | 56 | 9.2 | 811 | 70.1 | 6.5 | 538 | 65.1 | 7.7 |
| 20 | Cubital Tunnel Syndrome | 1305 | 59.7 | 8.4 | 2341 | 65.7 | 7.4 | 1921 | 61.4 | 8.3 |
| 21 | Cyclist's Palsy | 799 | 61.3 | 9.2 | 1892 | 74.5 | 6.5 | 1399 | 67.9 | 8.3 |
| 22 | De Quervain’s Tenosynovitis | 405 | 52.9 | 9.7 | 1014 | 70.1 | 6.8 | 698 | 62.1 | 8.2 |
| 23 | Dislocated Shoulder | 1635 | 49.1 | 10.7 | 3152 | 59 | 8.9 | 2487 | 53.3 | 10.1 |
| 24 | Distal Bicep Tendonitis | 791 | 59.1 | 9.3 | 1847 | 65.4 | 7.8 | 1338 | 63.1 | 8.5 |
| 25 | Dressings After Hand Injury or Surgery | 1298 | 68.5 | 8 | 2588 | 77.6 | 6.1 | 2099 | 73.5 | 7 |
| 26 | Dupuytren’s Contracture | 1759 | 60.8 | 8.2 | 3185 | 67.4 | 7.1 | 2303 | 62.3 | 7.9 |
| 27 | Edema Treatment for the Arm and Hand | 499 | 55.2 | 10.2 | 1203 | 76 | 5.9 | 918 | 66.4 | 8.1 |
| 28 | Elbow Dislocation | 1118 | 57.9 | 9 | 2116 | 63.3 | 8 | 1658 | 60.8 | 8.7 |
| 29 | Elbow Fracture | 991 | 55.9 | 9.8 | 2096 | 69.5 | 7.1 | 1538 | 64.1 | 8.3 |
| 30 | Elbow Pain: Stiffness | 1041 | 57.7 | 8.1 | 2541 | 67.7 | 6.8 | 1859 | 63.5 | 7.5 |
| 31 | Elbow Surgery: Arthroscopy | 538 | 48.8 | 10.1 | 1477 | 63 | 7.9 | 1042 | 57.2 | 9 |
| 32 | Extensor Tendon Injury | 922 | 63.8 | 8.1 | 1852 | 71.3 | 7 | 1368 | 67.8 | 7.6 |
| 33 | Fibromyalgia Hand Pain | 404 | 41.7 | 11.9 | 1029 | 54.8 | 9.3 | 697 | 49.3 | 10.6 |
| 34 | Flexor Tendon Injury | 576 | 53.2 | 10.3 | 1480 | 67.9 | 7.6 | 970 | 62.1 | 8.8 |
| 35 | Fractures in Children | 854 | 58 | 10.4 | 1945 | 74.9 | 6.7 | 535 | 81.6 | 5.9 |
| 36 | Frostbite in Hands | 1072 | 54.8 | 9.5 | 2335 | 68 | 7.2 | 1702 | 62 | 8.5 |
| 37 | Gamer’s/Texter’s Thumb | 757 | 49 | 12.5 | 1780 | 71.1 | 7.3 | 577 | 76.3 | 6.6 |
| 38 | Ganglion Cyst | 974 | 63.7 | 8.2 | 2101 | 74.6 | 6.4 | 1645 | 70.2 | 7.3 |
| 39 | Golf Injuries to the Hand, Wrist, or Elbow | 1456 | 47.4 | 12.3 | 2664 | 66.2 | 7.8 | 2099 | 55.2 | 10.4 |
| 40 | Gout in Hands | 609 | 67.6 | 7.1 | 1520 | 72.6 | 6.4 | 1004 | 70.5 | 6.8 |
| 41 | Hand Cramps (Focal Dystonia) | 691 | 54.9 | 9.3 | 1798 | 67.6 | 7.2 | 1237 | 62.7 | 8.3 |
| 42 | Hand Infection | 2135 | 46.5 | 11.3 | 4089 | 57.1 | 9.1 | 2923 | 51.1 | 10.3 |
| 43 | Hand Surgery Anesthesia | 967 | 49.8 | 10.4 | 2095 | 58.7 | 8.6 | 1568 | 54.7 | 9.6 |
| 44 | Hand Therapy | 640 | 53.1 | 8.6 | 1576 | 61.9 | 7.6 | 1089 | 55.9 | 8.6 |
| 45 | Hand Therapy for Hypersensitivity | 799 | 55.5 | 9.2 | 1817 | 67.4 | 7.4 | 1387 | 62.3 | 8.4 |
| 46 | Hand and Finger Exercises | 525 | 71 | 6.3 | 1445 | 80 | 4.8 | 989 | 76.3 | 5.5 |
| 47 | Hand or Wrist Joint Pain | 963 | 48.2 | 11.6 | 2054 | 62.9 | 8.1 | 1543 | 56.4 | 9.6 |
| 48 | Heat Treatment and Cold Treatment For Hands | 650 | 61.8 | 9.8 | 1506 | 72.8 | 6.8 | 1076 | 67.6 | 8.3 |
| 49 | How to Treat a Burn | 446 | 53.7 | 10.2 | 1216 | 76.6 | 6 | 822 | 68 | 7.7 |
| 50 | Jammed Finger | 363 | 59.2 | 8.7 | 1039 | 73.2 | 6.5 | 652 | 66.3 | 7.7 |
| 51 | Jersey Finger | 925 | 55.2 | 10 | 1959 | 68.1 | 7.3 | 533 | 70.9 | 7.3 |
| 52 | Kienbock's Disease | 505 | 56.1 | 9.3 | 1330 | 67.5 | 7.6 | 383 | 67.2 | 8.7 |
| 53 | Knuckle, Wrist & Joint Replacement | 713 | 58.2 | 8.9 | 1517 | 67.3 | 7.1 | 467 | 71 | 7.2 |
| 54 | MCP Joint Arthritis | 524 | 60.8 | 8.5 | 1229 | 72.3 | 6.3 | 375 | 73 | 7.2 |
| 55 | Mallet Finger | 1224 | 63.6 | 8.9 | 2111 | 72.6 | 6.9 | 397 | 76.7 | 6.5 |
| 56 | Nail Bed Injury | 224 | 57.6 | 9.6 | 644 | 71 | 7.1 | 182 | 75.1 | 6.6 |
| 57 | Nerve Damage and Repair | 676 | 66.3 | 8.7 | 1631 | 74.6 | 6.9 | 546 | 77.3 | 6.7 |
| 58 | Nerve Injury | 1279 | 66.5 | 8.4 | 2409 | 72 | 7 | 697 | 75.1 | 6.6 |
| 59 | Numbness in Hands | 1260 | 56.2 | 9.3 | 2348 | 65.2 | 7.6 | 611 | 69.2 | 7.5 |
| 60 | Olecranon Bursitis | 1058 | 56.4 | 9.3 | 2020 | 63.6 | 8 | 611 | 65.7 | 7.8 |
| 61 | Osteoarthritis | 620 | 61.4 | 8.8 | 1354 | 68.8 | 7.2 | 394 | 69.9 | 7.9 |
| 62 | Overuse Injuries | 926 | 54.1 | 9.7 | 1803 | 62.5 | 7.9 | 489 | 67.4 | 7.1 |
| 63 | Pain Management: How to Get Pain Relief | 951 | 59.7 | 9 | 2423 | 68 | 7.5 | 626 | 68.1 | 7.1 |
| 64 | Pain Medication: What are Opioids? | 606 | 55.9 | 8.9 | 1652 | 61.2 | 8 | 565 | 63 | 8.1 |
| 65 | Paraffin Wax Bath | 387 | 64.8 | 8.4 | 1081 | 77.3 | 6.2 | 341 | 80 | 6.3 |
| 66 | Pseudogout | 1131 | 55 | 8.8 | 2571 | 62.4 | 7.8 | 636 | 62.6 | 8.5 |
| 67 | Psoriatic Arthritis | 599 | 56 | 9.4 | 1428 | 60.5 | 8.2 | 403 | 60.1 | 8.4 |
| 68 | Radial Tunnel Syndrome | 672 | 49 | 10.1 | 1664 | 67.8 | 6.9 | 584 | 71.6 | 6.4 |
| 69 | Rheumatoid Arthritis | 786 | 52.4 | 9.2 | 1871 | 63.4 | 7.5 | 469 | 69.1 | 7.2 |
| 70 | Rock Climbing Injuries | 1809 | 50.7 | 10 | 3465 | 62.5 | 7.6 | 2598 | 55.4 | 9.1 |
| 71 | Rotator Cuff Injury | 1181 | 59.2 | 8.9 | 2413 | 64.7 | 7.8 | 724 | 66.2 | 7.9 |
| 72 | Scaphoid Fracture | 640 | 63.7 | 9.8 | 1637 | 79.4 | 6.2 | 422 | 81 | 6.2 |
| 73 | Scapholunate Torn Ligament | 838 | 60.1 | 8.4 | 2291 | 71.4 | 6.6 | 692 | 72.5 | 6.7 |
| 74 | Scar Treatment | 593 | 64.6 | 7.6 | 1431 | 74.5 | 6.2 | 446 | 78.7 | 5.8 |
| 75 | Shoulder Arthritis | 745 | 57.9 | 9.1 | 1639 | 63.2 | 8.1 | 553 | 62.5 | 8.6 |
| 76 | Shoulder Fracture | 461 | 60.4 | 8.7 | 1256 | 71.5 | 6.8 | 380 | 72.6 | 7 |
| 77 | Shoulder Pain | 775 | 54.7 | 9.2 | 1827 | 64.3 | 7.7 | 514 | 67.3 | 7.5 |
| 78 | Skiing and Snowboarding Injury Prevention | 869 | 70.5 | 7.3 | 1839 | 79.2 | 5.6 | 522 | 82.5 | 5.2 |
| 79 | Skin Cancer of the Hand and Upper Extremity | 1133 | 58.2 | 9.2 | 2449 | 68.6 | 7.1 | 700 | 71.3 | 7.1 |
| 80 | Splinters and Other Foreign Bodies in the Hand | 1735 | 58 | 9 | 2849 | 66 | 7.7 | 727 | 75.2 | 6.5 |
| 81 | Sprained Thumb | 367 | 71.6 | 7.1 | 976 | 80.6 | 5.4 | 295 | 82.9 | 5.5 |
| 82 | Sprained Wrist | 437 | 65 | 7.9 | 1131 | 77.5 | 5.6 | 341 | 79.3 | 5.5 |
| 83 | Stiff Hands | 763 | 55.3 | 8.5 | 1864 | 66.5 | 7.1 | 606 | 69.1 | 7.4 |
| 84 | Swan Neck Deformity | 321 | 58.1 | 9.8 | 948 | 72.7 | 7 | 293 | 73.8 | 7.8 |
| 85 | Swollen Fingers | 608 | 54.8 | 9.9 | 1390 | 68.2 | 7 | 393 | 74.1 | 6.3 |
| 86 | Systemic Disease | 715 | 53.1 | 10 | 1499 | 65.2 | 7.6 | 419 | 70.5 | 6.8 |
| 87 | TFCC Tear | 447 | 60.6 | 8.8 | 1104 | 71.4 | 7 | 379 | 74.4 | 6.6 |
| 88 | Tendon Transfer | 482 | 61.9 | 9.1 | 1283 | 72.3 | 6.6 | 408 | 76.9 | 5.9 |
| 89 | Tennis Elbow | 580 | 41.9 | 11.4 | 1413 | 60.8 | 8.2 | 464 | 64.5 | 8 |
| 90 | Thumb Arthritis | 520 | 50.9 | 10.2 | 1330 | 66.7 | 7.5 | 455 | 71.8 | 6.9 |
| 91 | Trigger Finger | 1346 | 58.5 | 9.2 | 2380 | 66.5 | 7.4 | 468 | 74.2 | 6.2 |
| 92 | Ulnar Wrist Disease | 1125 | 49.2 | 11.1 | 2538 | 63.6 | 7.9 | 802 | 68.1 | 7.1 |
| 93 | Vascular Disease | 904 | 58.5 | 9.6 | 1887 | 66.4 | 7.7 | 1420 | 63.3 | 8.3 |
| 94 | Warts on Hand | 296 | 64.1 | 7.9 | 811 | 73.2 | 6.2 | 239 | 70.8 | 6.4 |
| 95 | Wrist Arthritis | 821 | 55.1 | 9.1 | 1622 | 62.6 | 7.7 | 561 | 66.1 | 7.3 |
| 96 | Wrist Fracture | 1081 | 62.9 | 9.3 | 2212 | 74.4 | 6.4 | 550 | 80.1 | 5.5 |
| 97 | Wrist Surgery: Arthroscopy | 496 | 51.7 | 10 | 1257 | 67 | 7.3 | 433 | 70.8 | 6.8 |
| 98 | Wrist or Hand Tumor | 967 | 61.1 | 9.2 | 2135 | 70.2 | 7.1 | 665 | 74.6 | 6.3 |

**Supplemental Table S3.** Word count, FRE, and FKGL scores of original articles and those written at a 6^th^-grade or lower reading level by both ChatGPT and Google Gemini

|  |  | **Gemini: "Write patient education material over: "article title"** | | | **ChatGPT: "Write patient education material over "article title".** | | | **Gemini: "Write patient education materials over "article title" at a 6th grade or lower reading level."** | | | **ChatGPT: "Write patient education materials over "article title" at a 6th grade or lower reading level."** | | |
| --- | --- | --- | --- | --- | --- | --- | --- | --- | --- | --- | --- | --- | --- |
|  | **Article Title** | **Word Count** | **FRE** | **FKGL** | **Word Count** | **FRE** | **FKGL** | **Word Count** | **FRE** | **FKGL** | **Word Count** | **FRE** | **FKGL** |
| 1 | Amputation: Prosthetic Hand and Fingers | 681 | 38.9 | 11 | 798 | 41.2 | 11.2 | 463 | 75.8 | 5.5 | 2416 | 53 | 9.2 |
| 2 | Animal Bite | 626 | 50.5 | 9.7 | 679 | 15.1 | 9.2 | 380 | 77.8 | 5.1 | 348 | 74.5 | 5.7 |
| 3 | Antibiotics in Hand Surgery | 607 | 36.5 | 11 | 848 | 31.7 | 13 | 416 | 64.2 | 6.9 | 479 | 63.5 | 7 |
| 4 | Arm Cast and Splint Care | 636 | 65.9 | 7 | 667 | 61.4 | 8.3 | 527 | 88.9 | 3.6 | 360 | 85.4 | 4.7 |
| 5 | Arm, Hand, and Finger Replantation | 703 | 47.5 | 9.9 | 791 | 40.6 | 11.4 | 485 | 73.3 | 5.8 | 470 | 75.5 | 6.2 |
| 6 | Boutonniere Deformity | 572 | 44.2 | 9.9 | 819 | 44.5 | 11.6 | 414 | 81.2 | 4.7 | 444 | 73.2 | 6.5 |
| 7 | Boxer’s Fracture | 296 | 63.5 | 8.3 | 565 | 53.8 | 9.5 | 664 | 55.6 | 8.1 | 405 | 81.5 | 4.8 |
| 8 | Brachial Plexus Injury | 649 | 44.1 | 9.9 | 893 | 48 | 10.1 | 475 | 69.5 | 6.2 | 328 | 71 | 6 |
| 9 | Broken Arm | 725 | 61.7 | 8.2 | 779 | 62.5 | 7.8 | 565 | 88.1 | 3.8 | 471 | 87.8 | 4 |
| 10 | Broken Finger Tip | 553 | 55.2 | 8.7 | 716 | 54.6 | 9.2 | 467 | 89.7 | 2.8 | 426 | 82.9 | 4.4 |
| 11 | Broken Hand | 730 | 55.5 | 8.9 | 697 | 50.6 | 9.8 | 494 | 89.9 | 3.2 | 2311 | 97.5 | 7 |
| 12 | Carpal Boss | 592 | 57.3 | 8.3 | 677 | 46.9 | 11.4 | 463 | 88.5 | 3.5 | 418 | 82.4 | 4.9 |
| 13 | Carpal Tunnel Syndrome | 682 | 45.1 | 10.6 | 807 | 51.8 | 10 | 535 | 79.3 | 4.8 | 334 | 83.8 | 4.3 |
| 14 | Cigarettes and Hand Conditions | 485 | 48.9 | 10.5 | 798 | 50.3 | 10.7 | 319 | 83.5 | 4.6 | 385 | 87.2 | 4.3 |
| 15 | Cold Hands | 587 | 54.4 | 8.6 | 707 | 54.8 | 9.9 | 475 | 87.1 | 4.1 | 312 | 83.6 | 5.6 |
| 16 | Complex Regional Pain Syndrome - CRPS | 704 | 49.2 | 9.8 | 968 | 47.1 | 10.5 | 503 | 71.6 | 5.9 | 486 | 72.6 | 6.1 |
| 17 | Congenital Hand Differences | 543 | 42.7 | 10.6 | 706 | 33.8 | 12.8 | 484 | 69.3 | 6.2 | 522 | 65.5 | 8 |
| 18 | Congenital Trigger Thumb | 589 | 58.4 | 8.6 | 665 | 47 | 10.8 | 386 | 86.7 | 3.7 | 377 | 79.9 | 5.5 |
| 19 | Cortisone Shot | 678 | 44.1 | 10.3 | 682 | 47.6 | 10.5 | 405 | 84.4 | 3.9 | 475 | 80.2 | 5 |
| 20 | Cubital Tunnel Syndrome | 743 | 46.1 | 10.7 | 733 | 49.4 | 11 | 411 | 83.1 | 3.9 | 375 | 77.1 | 5.8 |
| 21 | Cyclist's Palsy | 673 | 50.7 | 9.7 | 674 | 50.1 | 10.2 | 455 | 85.8 | 4.2 | 417 | 84.5 | 5.4 |
| 22 | De Quervain’s Tenosynovitis | 630 | 50.8 | 9.4 | 723 | 50.7 | 10 | 445 | 85.5 | 3.8 | 419 | 72.8 | 6 |
| 23 | Dislocated Shoulder | 691 | 45.6 | 10.6 | 696 | 44.2 | 10.4 | 437 | 83.1 | 4.5 | 493 | 80.7 | 5.1 |
| 24 | Distal Bicep Tendonitis | 636 | 41.5 | 10.5 | 678 | 38.8 | 11.7 | 508 | 74.9 | 5.3 | 313 | 71.4 | 5.7 |
| 25 | Dressings After Hand Injury or Surgery | 648 | 54 | 8.8 | 793 | 59.1 | 8.4 | 410 | 90.9 | 3.1 | 445 | 86.2 | 4.3 |
| 26 | Dupuytren’s Contracture | 561 | 43.7 | 10.3 | 739 | 44 | 10.7 | 421 | 80.2 | 4.6 | 407 | 69.6 | 6.6 |
| 27 | Edema Treatment for the Arm and Hand | 583 | 43.9 | 9.9 | 786 | 52.1 | 9.9 | 442 | 89.2 | 2.7 | 441 | 82.5 | 4.5 |
| 28 | Elbow Dislocation | 507 | 54.4 | 8.9 | 675 | 49.1 | 10 | 476 | 82.8 | 3.9 | 380 | 78.9 | 5.1 |
| 29 | Elbow Fracture | 390 | 66.7 | 7.1 | 702 | 54.3 | 9.2 | 522 | 85.3 | 3.7 | 515 | 84.8 | 4.4 |
| 30 | Elbow Pain: Stiffness | 654 | 46.7 | 11.5 | 675 | 31.6 | 12.6 | 459 | 82.4 | 4 | 452 | 78.9 | 5 |
| 31 | Elbow Surgery: Arthroscopy | 712 | 38.2 | 11 | 771 | 40.7 | 11.4 | 516 | 80.7 | 4.6 | 490 | 67.8 | 7.1 |
| 32 | Extensor Tendon Injury | 609 | 53.9 | 8.9 | 832 | 42.4 | 11.3 | 491 | 84.2 | 3.9 | 501 | 72 | 6.5 |
| 33 | Fibromyalgia Hand Pain | 726 | 43.5 | 10.9 | 868 | 41.6 | 11.6 | 432 | 78.2 | 4.9 | 382 | 77 | 5.9 |
| 34 | Flexor Tendon Injury | 627 | 55.8 | 8.9 | 705 | 47.6 | 10.4 | 528 | 81.6 | 4.8 | 511 | 74.8 | 5.7 |
| 35 | Fractures in Children | 754 | 60.5 | 8 | 827 | 58.5 | 9.1 | 480 | 92.7 | 2.5 | 721 | 84.2 | 4.5 |
| 36 | Frostbite in Hands | 683 | 50.7 | 9 | 659 | 58.5 | 8.3 | 406 | 85.7 | 3.6 | 409 | 90.9 | 3.5 |
| 37 | Gamer’s/Texter’s Thumb | 613 | 58.1 | 8.4 | 805 | 48.6 | 10.8 | 447 | 91.5 | 2.7 | 414 | 85.9 | 4.7 |
| 38 | Ganglion Cyst | 619 | 60.8 | 7.9 | 562 | 52.2 | 9.8 | 453 | 87.9 | 3.5 | 437 | 80.5 | 4.7 |
| 39 | Golf Injuries to the Hand, Wrist, or Elbow | 631 | 47.1 | 10 | 791 | 50.7 | 10 | 427 | 85.7 | 4 | 461 | 82.1 | 5.2 |
| 40 | Gout in Hands | 715 | 51.8 | 9.6 | 814 | 53.8 | 9.1 | 507 | 82.4 | 4.5 | 431 | 84.2 | 4.5 |
| 41 | Hand Cramps (Focal Dystonia) | 726 | 40.2 | 11 | 747 | 35.3 | 12.3 | 431 | 80.6 | 4.5 | 361 | 83.4 | 4.8 |
| 42 | Hand Infection | 607 | 42.2 | 10.1 | 777 | 49.8 | 10.3 | 416 | 86.4 | 3.3 | 440 | 80.2 | 4.7 |
| 43 | Hand Surgery Anesthesia | 651 | 37.3 | 11.7 | 898 | 42.3 | 12 | 518 | 72.2 | 6 | 467 | 69.5 | 7.3 |
| 44 | Hand Therapy | 518 | 72.2 | 6 | 637 | 37.3 | 12.1 | 415 | 79.1 | 5.2 | 462 | 84.2 | 4.6 |
| 45 | Hand Therapy for Hypersensitivity | 589 | 48.2 | 9.7 | 695 | 35.8 | 11.8 | 470 | 73.6 | 5.7 | 452 | 71.3 | 6.8 |
| 46 | Hand and Finger Exercises | 568 | 59.9 | 7.4 | 750 | 56.2 | 8.8 | 377 | 85.7 | 3.1 | 432 | 80.1 | 5.1 |
| 47 | Hand or Wrist Joint Pain | 773 | 51 | 9.9 | 703 | 48.3 | 10.2 | 522 | 82.5 | 4.4 | 473 | 81.4 | 5.3 |
| 48 | Heat Treatment and Cold Treatment For Hands | 630 | 52.2 | 9.3 | 683 | 56.8 | 8.7 | 524 | 80.8 | 4.7 | 455 | 87.9 | 3.5 |
| 49 | How to Treat a Burn | 648 | 55.1 | 8.4 | 675 | 60 | 7.6 | 430 | 83.3 | 3.9 | 355 | 81.5 | 5 |
| 50 | Jammed Finger | 606 | 55.2 | 8.5 | 717 | 52.7 | 10.3 | 422 | 87.6 | 2.9 | 290 | 83.9 | 4.8 |
| 51 | Jersey Finger | 521 | 54.8 | 8.8 | 661 | 53.9 | 9.8 | 443 | 83.7 | 4.4 | 293 | 81.8 | 4.5 |
| 52 | Kienbock's Disease | 666 | 51 | 9.2 | 904 | 51.7 | 10.1 | 416 | 84.5 | 4.4 | 506 | 78.7 | 5.7 |
| 53 | Knuckle, Wrist & Joint Replacement | 771 | 38.5 | 11.4 | 929 | 35.9 | 12.7 | 445 | 78.7 | 4.8 | 568 | 75.2 | 5.8 |
| 54 | MCP Joint Arthritis | 653 | 40.9 | 11.2 | 712 | 48 | 10.4 | 426 | 80.3 | 4.5 | 348 | 69.2 | 7 |
| 55 | Mallet Finger | 584 | 55.9 | 8.6 | 647 | 51.6 | 9.6 | 411 | 89.4 | 3.3 | 407 | 81.4 | 5.2 |
| 56 | Nail Bed Injury | 653 | 62.9 | 7.5 | 730 | 60.1 | 8.4 | 407 | 94.4 | 2.6 | 378 | 86.3 | 3.9 |
| 57 | Nerve Damage and Repair | 706 | 44.1 | 10 | 845 | 38.5 | 11.6 | 385 | 78.1 | 4.6 | 433 | 80.5 | 4.8 |
| 58 | Nerve Injury | 626 | 47.2 | 9.9 | 673 | 46.6 | 10 | 406 | 79.8 | 4.5 | 405 | 81.9 | 4.5 |
| 59 | Numbness in Hands | 683 | 49.6 | 9.4 | 737 | 49.2 | 10.9 | 451 | 87 | 3.8 | 385 | 83.4 | 6 |
| 60 | Olecranon Bursitis | 650 | 43.8 | 10.2 | 744 | 43.9 | 11.7 | 486 | 77.5 | 4.8 | 323 | 70.1 | 6.5 |
| 61 | Osteoarthritis | 755 | 53.6 | 9.1 | 692 | 52.5 | 9.1 | 586 | 65.1 | 7.2 | 323 | 68.8 | 6.6 |
| 62 | Overuse Injuries | 647 | 41.6 | 10.2 | 670 | 40.7 | 11.5 | 516 | 79.9 | 4.6 | 374 | 78.5 | 4.7 |
| 63 | Pain Management: How to Get Pain Relief | 677 | 41.8 | 11 | 753 | 34.1 | 13.1 | 398 | 80.2 | 4.1 | 485 | 80.3 | 5.5 |
| 64 | Pain Medication: What are Opioids? | 573 | 46.1 | 10 | 692 | 38.1 | 11.9 | 422 | 66 | 6.6 | 487 | 74 | 5.7 |
| 65 | Paraffin Wax Bath | 555 | 60.3 | 7.8 | 751 | 58.4 | 9.1 | 455 | 89.2 | 4 | 433 | 87.5 | 4.6 |
| 66 | Pseudogout | 630 | 46 | 9.9 | 726 | 42.3 | 11.2 | 443 | 82.2 | 4.2 | 410 | 76.1 | 5.2 |
| 67 | Psoriatic Arthritis | 718 | 40.3 | 10.9 | 699 | 46.6 | 9.8 | 477 | 73.7 | 5.3 | 406 | 71.8 | 6.7 |
| 68 | Radial Tunnel Syndrome | 651 | 43.4 | 11 | 711 | 42.5 | 11.5 | 529 | 79.5 | 4.8 | 337 | 76.8 | 5.7 |
| 69 | Rheumatoid Arthritis | 709 | 46.2 | 9.6 | 807 | 42.4 | 11.3 | 443 | 82.7 | 4.3 | 613 | 74 | 6.1 |
| 70 | Rock Climbing Injuries | 698 | 49.4 | 9.5 | 720 | 41.8 | 10.5 | 470 | 77.3 | 5.3 | 488 | 79.7 | 5.4 |
| 71 | Rotator Cuff Injury | 694 | 40.3 | 10.8 | 604 | 40.3 | 11.5 | 502 | 75.8 | 5.4 | 366 | 75.5 | 6.2 |
| 72 | Scaphoid Fracture | 624 | 59.4 | 7.8 | 729 | 58.4 | 9.3 | 418 | 82.6 | 4.5 | 397 | 86.3 | 4.1 |
| 73 | Scapholunate Torn Ligament | 628 | 41.5 | 11.1 | 821 | 43.2 | 11.4 | 421 | 84.9 | 3.7 | 427 | 85.3 | 4.2 |
| 74 | Scar Treatment | 682 | 52 | 9.5 | 716 | 53.1 | 9.4 | 510 | 81.7 | 4.6 | 389 | 82.9 | 4.7 |
| 75 | Shoulder Arthritis | 695 | 39.3 | 11.2 | 781 | 42.7 | 11.1 | 461 | 73.5 | 6 | 517 | 67.9 | 6.7 |
| 76 | Shoulder Fracture | 672 | 51.5 | 9.4 | 716 | 45.2 | 10 | 425 | 87.9 | 3.3 | 508 | 85 | 4.4 |
| 77 | Shoulder Pain | 610 | 43.9 | 10 | 776 | 43.7 | 10.9 | 509 | 74.1 | 5.4 | 505 | 72.4 | 6.2 |
| 78 | Skiing and Snowboarding Injury Prevention | 581 | 52.1 | 9 | 813 | 61.2 | 8.1 | 446 | 77.9 | 4.9 | 398 | 77.3 | 5.1 |
| 79 | Skin Cancer of the Hand and Upper Extremity | 691 | 50.6 | 9.7 | 862 | 59.7 | 8.1 | 499 | 87.1 | 4 | 437 | 82 | 4.8 |
| 80 | Splinters and Other Foreign Bodies in the Hand | 542 | 63.5 | 7.3 | 685 | 56.9 | 8.8 | 420 | 83.6 | 4.1 | 474 | 84 | 4.6 |
| 81 | Sprained Thumb | 622 | 55.4 | 9.1 | 547 | 61.4 | 8 | 420 | 90.2 | 3.4 | 404 | 85.5 | 4.6 |
| 82 | Sprained Wrist | 668 | 58 | 8.2 | 573 | 51.6 | 9.2 | 393 | 91 | 2.7 | 298 | 84.6 | 4 |
| 83 | Stiff Hands | 664 | 52.4 | 9.4 | 659 | 50.3 | 10.3 | 475 | 91.6 | 4 | 367 | 90.5 | 3.8 |
| 84 | Swan Neck Deformity | 675 | 41.4 | 10.5 | 816 | 41 | 11.4 | 384 | 79.1 | 4.8 | 346 | 76.4 | 5.4 |
| 85 | Swollen Fingers | 647 | 45.8 | 10.6 | 618 | 40.2 | 11.7 | 429 | 81.3 | 5.1 | 408 | 75.1 | 5.9 |
| 86 | Systemic Disease | 624 | 38.2 | 11.1 | 701 | 38.1 | 11.7 | 402 | 79.3 | 4.7 | 378 | 67.6 | 7 |
| 87 | TFCC Tear | 633 | 45.5 | 10 | 708 | 55.7 | 8.8 | 429 | 91.3 | 2.7 | 381 | 81.1 | 4.9 |
| 88 | Tendon Transfer | 707 | 40.7 | 11 | 802 | 36.7 | 15.2 | 383 | 87.5 | 3.4 | 429 | 76.1 | 5.8 |
| 89 | Tennis Elbow | 623 | 49.2 | 9.3 | 676 | 43.8 | 10.9 | 499 | 77.7 | 4.7 | 449 | 75 | 6.2 |
| 90 | Thumb Arthritis | 697 | 47.3 | 10 | 745 | 47.3 | 10.5 | 459 | 83.3 | 4.3 | 343 | 75.2 | 5.7 |
| 91 | Trigger Finger | 617 | 49.1 | 9.6 | 743 | 13.3 | 11 | 412 | 83.6 | 4.3 | 372 | 71.6 | 6.2 |
| 92 | Ulnar Wrist Disease | 635 | 48 | 9.7 | 834 | 38.9 | 12.4 | 545 | 80.8 | 4.6 | 353 | 78.3 | 5.8 |
| 93 | Vascular Disease | 764 | 46 | 10 | 966 | 47.9 | 10.3 | 581 | 77 | 5.2 | 396 | 82.1 | 4.6 |
| 94 | Warts on Hand | 696 | 56.5 | 8.4 | 748 | 64.2 | 7.8 | 431 | 80.5 | 4.5 | 341 | 84 | 4.4 |
| 95 | Wrist Arthritis | 758 | 48.6 | 9.9 | 736 | 46.5 | 10.2 | 519 | 81.2 | 4.5 | 417 | 70.2 | 6.6 |
| 96 | Wrist Fracture | 614 | 55.1 | 9.1 | 749 | 61.3 | 8.2 | 506 | 93.3 | 2.8 | 419 | 86.5 | 4.2 |
| 97 | Wrist Surgery: Arthroscopy | 717 | 43.1 | 10.5 | 751 | 53.6 | 11.1 | 489 | 82 | 4 | 452 | 76.7 | 5.5 |
| 98 | Wrist or Hand Tumor | 711 | 56.4 | 8.5 | 998 | 50.3 | 10.6 | 518 | 84.5 | 4.6 | 678 | 80.9 | 5.2 |
